# A rare cause of adult ileocolic intussusception: ileal leiomyoma

**DOI:** 10.1259/bjrcr.20170094

**Published:** 2018-05-24

**Authors:** Kevin Z Zhou, Marcela Mautone, Parm Naidoo

**Affiliations:** 1 Diagnostic Imaging Department, Monash Health, Melbourne, VIC, Australia; 2 Department of Surgery, University of Melbourne, Melbourne, VIC, Australia; 3 Southern Clinical School, Monash University, Melbourne, VIC, Australia

## Abstract

Intussusception is a rare condition in adulthood and, unlike in children, is usually caused by an identifiable underlying lesion, most commonly a gastrointestinal tumour. The clinical presentation is non-specific and often there are intermittent symptoms making the diagnosis difficult based solely on history and examination. Plain radiography may reveal signs of bowel obstruction, however, CT is the gold standard to diagnose and localise an intussusception in adults. We present an unusual case of adult ileocolic intussusception caused by an ileal leiomyoma. This case highlights the important radiological findings of intussusception presenting with a high-grade obstruction and discusses the potential causes which should be considered.

## Case Presentation

A 50-year-old female with no significant past medical history presented to the emergency department of a tertiary hospital with a 5-day history of intermittent lower abdominal pain which had worsened in the preceding few hours. The pain was associated with nausea, vomiting and anorexia. She had been constipated for 4 days which was atypical for the patient. On physical examination, the abdomen was distended, guarded and tender to percussion and on rebound. Bowel sounds were hyperactive and a PR examination was unremarkable. Blood tests showed mild hyponatraemia and acute kidney injury but there was no elevation in lactate to suggest ischaemia. White cell count, liver function tests and other electrolytes were unremarkable. She was hemodynamically stable and proceeded to have a contrast-enhanced CT of the abdomen and pelvis with contrast.

## Investigations

**Figure 1.**
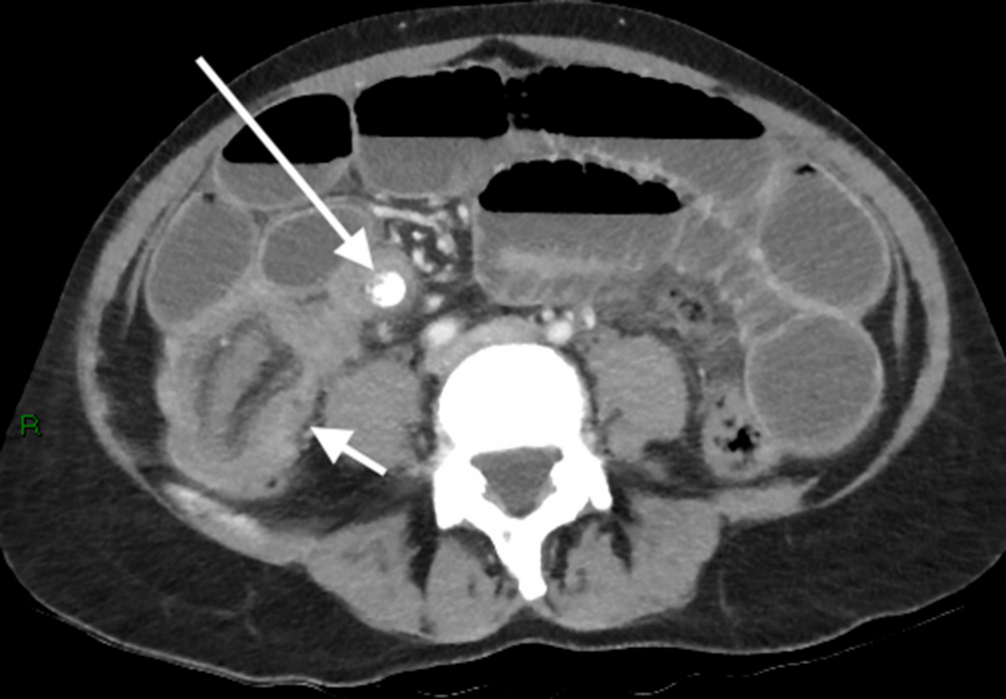
Axial and coronal images demonstrate several dilated loops of small bowel and a dilated stomach, compatible with high-grade obstruction. The obstruction extends to a 12 mm calcified mass (long arrow) within the lumen of the terminal ileum. The ileum at this level has a thickened wall and is collapsed distally. Telescoping of the collapsed ileum into the caecum (short arrow) is demonstrated, consistent with ileocolic intussusception.

**Figure 2.**
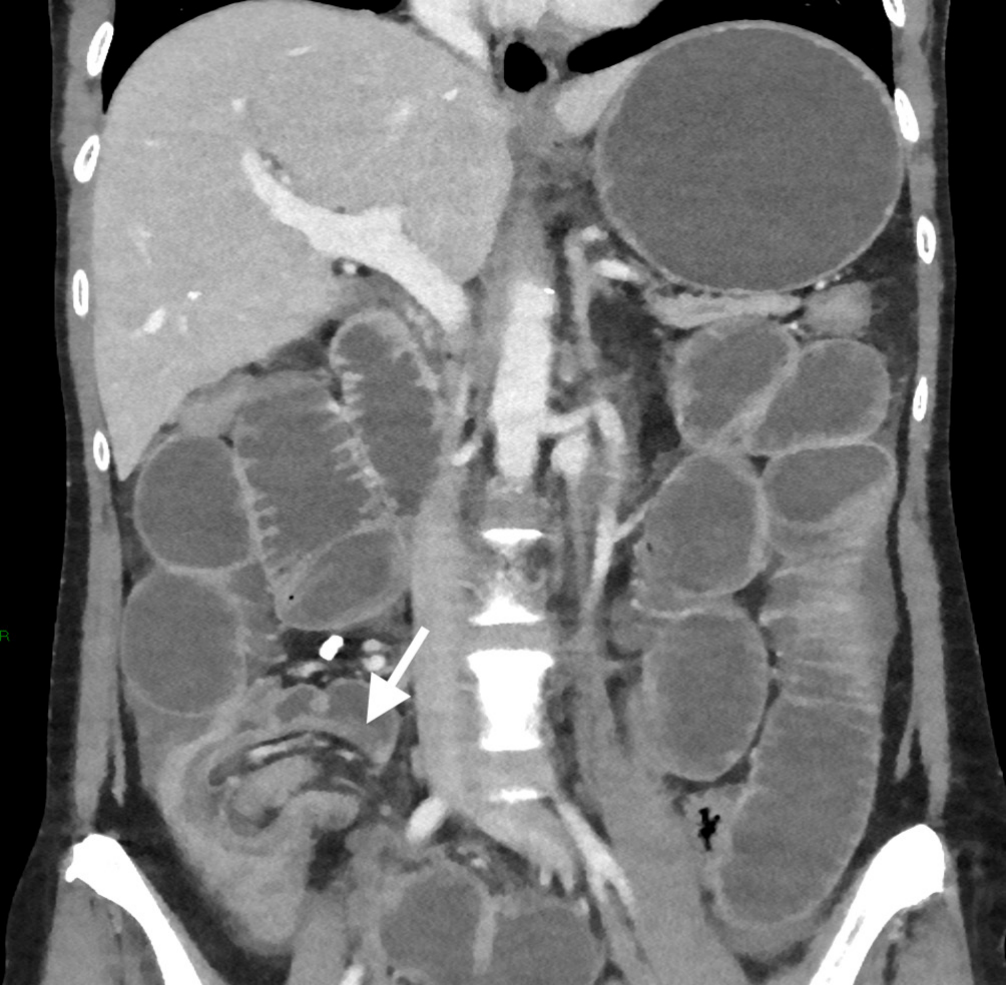
The ileum at this level has a thickened wall and is collapsed distally. Telescoping of the collapsed ileum into the caecum (short arrow) is demonstrated, consistent with ileocolic intussusception.

**Figure 3.**
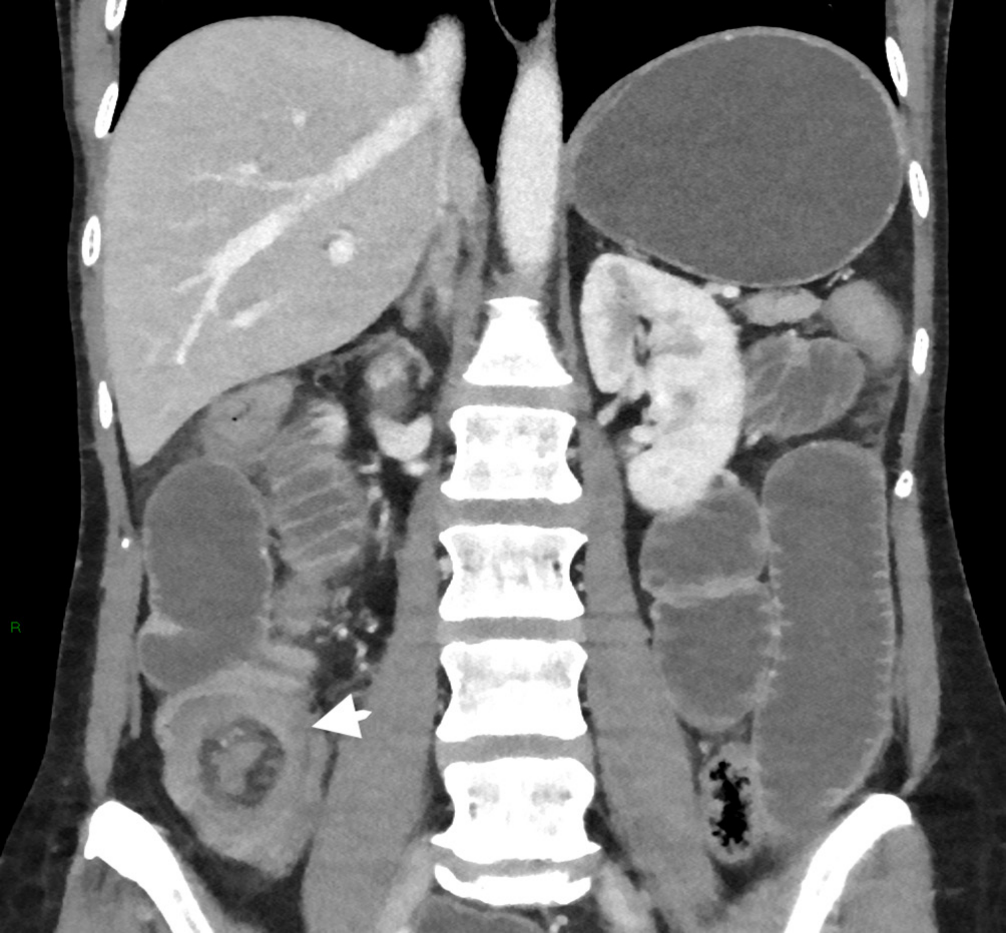
The caecum is also thick walled, suspicious for venous ischaemia. Note the bowel-within-bowel configuration of the ileocolic intussusception (arrowhead).

Abdominal CT with intravenous contrast showed marked fluid distension of the stomach and small bowel, in keeping with a high-grade obstruction, which extended to a 12 mm calcified lesion in the terminal ileum (Figure 1). There was a typical appearance of terminal ileum telescoping into the caecum at the point of the lesion and distally the colon was completely collapsed (Figure 2), consistent with an ileocolic intussusception with the calcified mass as the lead point. The wall of the distal ileum surrounding the lesion and the caecum was oedematous and thickened but there was no sign of pneumatosis intestinalis or pneumoperitoneum (Figure 3).

## Differential Diagnosis

The aetiology of the calcified mass was not apparent on imaging. A gallstone ileus was thought possible, given its dense calcification, however, there was no pneumobilia to support this differential diagnosis. Other differentials included an ingested foreign body, or calcified polyp or tumour such as a gastrointestinal stromal tumour or adenocarcinoma.

## Outcome and Follow-up

The patient proceeded to have laparotomy, which confirmed a 15 cm ileocolic intussusception with ischaemic changes noted in the bowel and near perforation of the terminal ileum. A routine right hemicolectomy was performed with primary anastomosis between the viable ileum and the transverse colon. The patient’s post-operative recovery was complicated by a brief ileus which resolved and she was discharged from the hospital 5 days later.

Pathological examination of the resected bowel showed an 18 × 12 × 15 mm hyalinized nodule with irregular, dystrophic calcification. Spindle cells focally involved the outer aspects and these stained with actin and desmin, confirming the lesion to be a submucosal leiomyoma.

## Discussion

Intussusception in adults is rare, accounting for less than 5% of bowel obstructions, but can be an often missed surgical emergency and indicator of underlying pathology.^1^ Adults tend to present with intermittent or vague abdominal pain over a period of time without any distinct clinical signs on examination to differentiate it from other causes of obstruction.^1^ The diagnosis is made through either radiological findings, laparoscopic examination, or intraoperatively during the laparotomy. An underlying cause of intussusception is found in more than 90% of cases; because of this, radiological investigation and surgical intervention are almost always indicated in adults.^1^

Plain abdominal radiographs can reveal distended loops of bowel and air–fluid levels, which are typical of bowel obstruction, but are generally unable to discern the cause of the blockage.^2^ Importantly, subdiaphragmactic air, if present, can be seen indicating a potential perforation of the bowel. On ultrasonography, the characteristic findings are a “target sign when viewing the bowel in a transverse plane and “pseudokidney” sign when viewing it in the longitudinal plane.^2^ Ultrasound is a reliable diagnostic tool, especially in children, with the added benefit of being radiation free.^1–3^

CT is generally accepted as the most sensitive and specific radiological investigation for intussusception and is the modality of choice in adults.^1–3^ It is also frequently used as an investigative tool for undifferentiated abdominal pain which is often how intussusception presents.^3^ Like ultrasound, the exact appearance is dependent on the plane the images are taken. A bowel-within-bowel configuration forming a series of concentric rings, much like the target sign, is typical when the CT image is perpendicular to the bowel.^2, 4^ In a longitudinal axis, the image is akin to a sausage.^2, 4^ A CT scan of the abdomen and pelvis also provides the radiologist with significant amounts of other important information. The location and extent of the intussusception can be characterized, along with the presence of any ischaemia or perforation, greatly aiding initial management and surgical planning. The lead point, if present, can usually be identified suggesting the nature of the underlying cause. Finally, in the event malignancy is suspected, assessment for locoregional metastases can be performed.

An underlying pathological cause for intussusception can be identified in more than 90% of adults.^1–4^ The most common cause is a neoplastic process, either benign or malignant, but rarer causes include Meckel’s diverticula, strictures, adhesions and a single case report of a gallstone causing secondary intussusception.^1, 5^ The location of the intussusception may suggest the nature of the tumour; with those occurring in the colon most often being malignant and those in the small bowel predominantly benign.^1^ Leiomyomas are the most common benign tumour of the small bowel. Other benign tumours which could act as a lead point in intussusception include inflammatory fibroid polyps and gastrointestinal stromal tumours.^6, 7^ A search of the English-language literature found six previous reported cases of a leiomyoma causing intussusception with five of them being jejunojejunal and one being duodenojejunal.^8–13^ All were diagnosed on CT with a visible lead point while plain radiography, ultrasound and endoscopy served as adjuncts. Leiomyomas were subsequently confirmed on histopathology. Neoplasia should always be suspected in adult intussusception and radiological assessment should be undertaken for metastatic disease, especially in a colocolic intussusception.

## Learning Points

1. The case highlights an uncommon but noteworthy cause of bowel obstruction in adults: intussusception.
2. An underlying pathological cause can be identified in more than 90% of cases of adult intussusception. A malignant tumour is the most common cause when the lead point originates from the large bowel. A benign tumour is more common when it originates from the small bowel.
3. CT is the investigation of choice to both diagnose the intussusception and assess the cause, location, extent and potential spread of a tumour.
